# Nifuroxazide exerts potent anti-tumor and anti-metastasis activity in melanoma

**DOI:** 10.1038/srep20253

**Published:** 2016-02-02

**Authors:** Yongxia Zhu, Tinghong Ye, Xi Yu, Qian Lei, Fangfang Yang, Yong Xia, Xuejiao Song, Li Liu, Hongxia Deng, Tiantao Gao, Cuiting Peng, Weiqiong Zuo, Ying Xiong, Lidan Zhang, Ningyu Wang, Lifeng Zhao, Yongmei Xie, Luoting Yu, Yuquan Wei

**Affiliations:** 1 State Key Laboratory of Biotherapy/ Collaborative Innovation Center for Biotherapy, West China Hospital, West China Medical School, Sichuan University, Chengdu, 610041, Sichuan, China; 2 College of agricultural and life sciences, University of Wisconsin-Madison, Madison, WI53706, USA; 3 Department of Pharmaceutical and Bioengineering, School of Chemical Engineering, Sichuan University, Chengdu, 610041, Sichuan, China; 4 Department of Pharmacy, Xinqiao Hospital, Third Military Medical University, Chongqing, 404100, China

## Abstract

Melanoma is a highly malignant neoplasm of melanocytes with considerable metastatic potential and drug resistance, explaining the need for new candidates that inhibit tumor growth and metastasis. The signal transducer and activator of the transcription 3 (Stat3) signaling pathway plays an important role in melanoma and has been validated as promising anticancer target for melanoma therapy. In this study, nifuroxazide, an antidiarrheal agent identified as an inhibitor of Stat3, was evaluated for its anti-melanoma activity *in vitro* and *in vivo*. It had potent anti-proliferative activity against various melanoma cell lines and could induce G2/M phase arrest and cell apoptosis. Moreover, nifuroxazide markedly impaired melanoma cell migration and invasion by down-regulating phosphorylated-Src, phosphorylated-FAK, and expression of matrix metalloproteinase (MMP) -2, MMP-9 and vimentin. It also significantly inhibited tumor growth without obvious side effects in the A375-bearing mice model by inducing apoptosis and reducing cell proliferation and metastasis. Notably, nifuroxazide significantly inhibited pulmonary metastases, which might be associated with the decrease of myeloid-derived suppressor cells (MDSCs). These findings suggested that nifuroxazide might be a potential agent for inhibiting the growth and metastasis of melanoma.

Malignant melanoma is one of the deadliest form of skin cancers, and the incidence is increasing rapidly compared to of any other solid tumors^1^,^2^. In 2014, around 73,870 new cases melanoma were diagnosed in the United States, accounting for 4.6% of new cases^3^. stage Patients with stage IV melanoma, which has spread to the lymph nodes or other organs, had a survival of just 8–9 months, and only 15% could live for more than 3 years^4^. In 2011 and 2013, the US Food and Drug Administration (FDA) approved small-molecule inhibitors vemurafenib (Zelboraf) and dabrafenib (Tafinlar) for use in people with the B-RAF (V600E) mutation, respectively^5^,^6^,^7^. Although response rates are high and rapid, cancer is a wily foe, and many people who took vemurafenib developed resistance within six months^8^. Besides, these available drugs also promoted invasiveness and metastasis of resistant cells^9^. High-metastatic potential and a high frequency of drug resistance have resulted in poor efficacy against metastatic melanoma^10^,^11^,^12^. Thus, new candidates for metastatic melanoma that exert potential anti-tumor activity and low toxicity are urgently needed.

Many signaling pathways are involved in melanoma progression and metastasis, including signal transducer and activator of transcription 3 (Stat3) signaling. Stat3 protein is constitutively activated in many tumor cells, including myeloma, breast cancer, lung cancer, and melanoma, but is not required for the function of most normal cells^13^,^14^. Stat3 plays a critical role in the regulation of fundamental biological processes, such as cell proliferation, apoptosis, angiogenesis, invasion and metastasis^15^,^16^,^17^,^18^. Besides, Stat3 can also lead to suppression of the innate and adaptive immune response by mediating accumulation of myeloid derived suppressor cells (MDSCs) in tumor^19^,^20^,^21^. In case of melanoma, the expression of phosphorylated Stat3 (p-Stat3) has been shown to be high and is associated with melanoma progression and/or development^18^,^22^. Importantly, Stat3 activity increased melanoma invasiveness and is required for active melanogenesis by regulating tyrosinase gene expression and enzyme activity^23^. Moreover, increasing evidences demonstrated that blockade of Stat3 by inhibitors could inhibit angiogenesis and trigger apoptosis in melanoma cells^24^,^25^, and inhibit cellular growth in melanoma cells with acquired resistance to vemurefenib^26^. These data all suggest that Stat3 inhibition provides a rational approach to therapy for melanoma.

Drug discovery is an expensive and time-consuming process, and one solution is to find new uses for the existing drugs^27^. Because of the known pharmacokinetics and safety profiles, the old drugs could be approved by regulatory agencies for human use easily^28^. Nifuroxazide is an oral nitrofuran antibiotic that decreases the viability of multiple myeloma cells and breast cancer cells by inhibiting the phosphorylation of Stat3^16^,^29^. Considering the effects of Stat3 in melanoma, we hypothesized that nifuroxazide, a potent inhibitor of Stat3, might be useful in the treatment of patients with melanoma.

To verify this hypothesis, this study attempts to evaluate the biological activities of nifuroxazide in melanoma *in vitro* and *in vivo*. Our data provided evidence that nifuroxazide could inhibit the proliferation of melanoma cells by inducing cell cycle arrest via inhibition of CDK1 and cyclin B1, and by inducing apoptosis via the reactive oxygen species (ROS)-mitochondria apoptotic pathway. We also found that nifuroxazide could suppress cell migration and invasion. Moreover, we evaluated the antitumor activity of nifuroxazide in A375 tumor-bearing xenograft mice model and B16-F10 melanoma metastasis model. These data suggested that nifuroxazide could be a new agent for the treatment of melanoma.

## Results

### Nifuroxazide inhibited cell proliferation and induced G2/M phase arrest in melanoma cells

**Figure 1:**
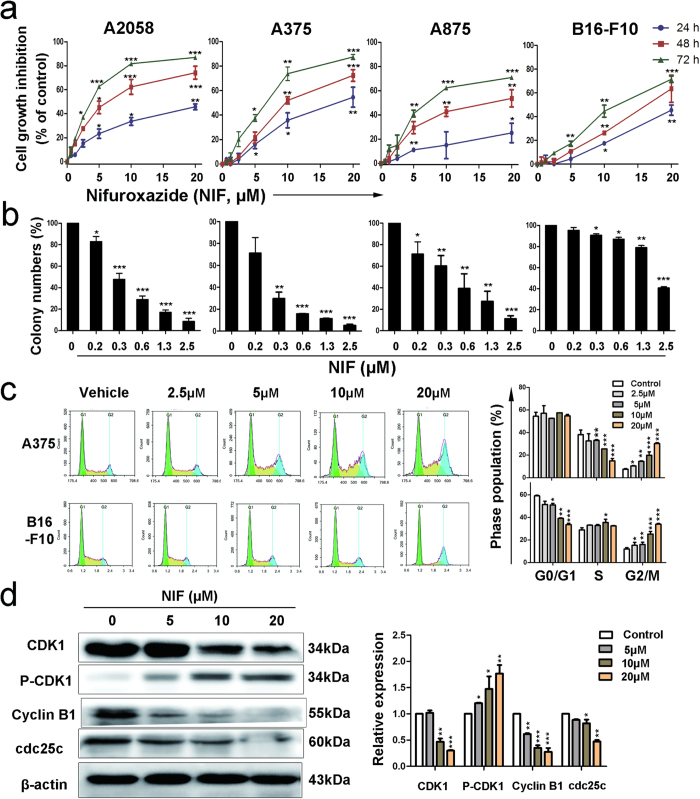
Nifuroxazide reduced viability and induced G2/M phase arrest in melanoma cells. (**a**) Melanoma cell lines A2058, A375, A875 and B16-F10 were treated with different concentrations of nifuroxazide for 24, 48 or 72 h and cell viability was measured by the MTT assay. Each point represents the mean ± SD for at least 3 independent experiments (*P < 0.05; **P < 0.01; ***P < 0.001 vs vehicle control). (**b**) The percentage of inhibition in colony-forming assays was expressed using vehicle treated cells at 100%. (**c**) A375 and B16-F10 cells were incubated with nifuroxazide for 48 h, and subjected to cell cycle analysis by FCM after incubated with a PI solution. The cell cycle distributions were displayed in quantified histograms. (**d**) XX induced G2/M phase arrest of A375 cells through down-regulation of CDK1-cyclin B1 complex activity. After exposure of A375 cells to the indicated concentrations of nifuroxazide (0, 5, 10, 20 μM) for 24 h, the protein levels of CDK1-cyclin B1complex, activity-related CDK1, cyclin B1, P-CDK1 (Tyr15) and cdc25c were determined by western blot with special antibodies, and protein expressions were quantified.

To evaluate the effects of nifuroxazide on melanoma, A2058, A375, A875 and B16-F10 cell lines were used in this study. Firstly, the levels of phosphorylated Stat3 (Tyr705) in four melanoma cell lines and one non-cancerous cell line (HEK293) were analyzed by western blot analysis. The results indicated that most melanoma cancer cells had constitutively activated Stat3 as assessed by its phosphorylation status at Tyr705, while the HEK293 cells almost no expression of p-Stat3 (Supplementary Fig. S1a). Then, we investigated the time- and concentration-dependent effects of nifuroxazide on melanoma cells. As shown in Fig. 1a, exposure of cells to various concentrations of nifuroxazide for 24 h, 48 h and 72 h resulted in decreased cell growth at increasing concentration and duration of exposure. Moreover, colony formation assay was performed to visually assess the anti-viability activity of nifuroxazide. Obviously, the size of the colony formation of melanoma cells treated with nifuroxazide was significantly smaller than the control group (Fig. 1b and Supplementary Fig. S1b).

To examine whether the anti-viability activity of nifuroxazide in melanoma cells was associated with cell cycle arrest, the cell lines A375 and B16-F10 were exposed to nifuroxazide at concentrations ranging from 0 to 20 μM for 24 h or 48 h, and cell cycle distribution was analyzed by flow cytometry (FCM). As shown in Fig. 1c and Supplementary Fig. S2, treatment with nifuroxazide for 24 h and 48 h induced significant G2/M phase arrest in a concentration-dependent manner in both A375 and B16-F10 cells. Compared with the G2/M phase distribution of the vehicle control group (11.9%), the percentage of G2/M fraction increased to 12.3%, 16.0%, 25.3%, and 33.9% in A375 cells treated with 2.5, 5, 10, and 20 μM nifuroxazide for 48 h, respectively. Similar results were observed for B16-F10 cells after nifuroxazide treatment (Fig. 1c).

To further elucidate the mechanisms, we investigated the expression levels of some key proteins involved in the G2/M phase transition by western blot in A375 cell line. The results showed that nifuroxazide decreased the expressions of cyclin B1, CDK1 and cdc25c and increased the phosphorylation status of CDK1 (Tyr15) in a dose-dependent manner (Fig. 1d), indicating that nifuroxazide inactivated the CDK1/cyclin B1 complex, which plays an important role during the transition from G2 to M phase. The above data indicated that nifuroxazide induced G2/M arrest via the inhibition of the cyclin B1/CDK1 complex and cdc25c.

### Nifuroxazide induced apoptosis in melanoma cells

We next explored whether nifuroxazide induced melanoma cell apoptosis. Hoechst 33358 staining assay showed that nifuroxazide altered the morphology of A375 cells after 24 h treatment and resulted in nuclear fragmentation, cell shrinking and formation of condensed nuclei with bright-blue fluoresce. Besides, these changes were concentration-dependent (Supplementary Fig. S3a). The morphologic changes were also observed in B16-F10 cells (Supplementary Fig. S3a).

**Figure 2:**
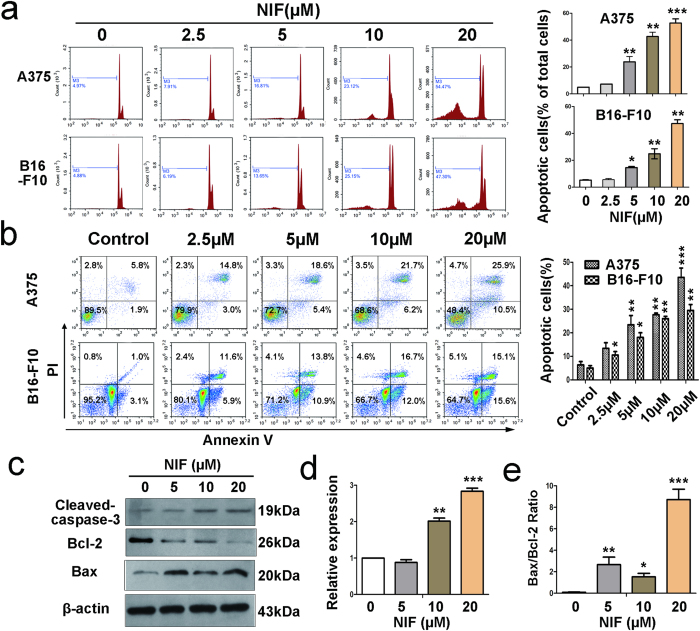
Nifuroxazide induced apoptosis of A375 and B16-F10 cells. (**a**) A375 and B16-F10 cells were treated with indicated concentrations of nifuroxazide for 48 h, respectively, and then were analyzed by FCM after PI-staining. Quantified values of the apoptosis were shown on the right. Each point represents the mean ± SD for at least 3 independent experiments (*P < 0.05; **P < 0.01; ***P < 0.001 vs vehicle control). The apoptosis of A375 (**b**) and B16-F10 (**c**) was analyzed using the Annexin V-FITC/PI dual-labeling technique. The proportions of apoptotic cells, including the early apoptotic cells and the late apoptotic cells, were both shown on the right. (**d**) Western blot was used to detect the levels of the typical apoptosis-related proteins cleaved caspase-3 and Bcl-2 family proteins. After treated with nifuroxazide, the expressions of cleaved caspase-3, Bcl-2, and Bax were determined by western blot with the expression of β-actin as the internal control. (**e**) Protein expression was quantified and normalized against β-actin expression. (**f**) The percentage of Bax/Bcl-2 ratio was presented in the bar graphs.

Further, we analyzed the apoptotic effects of nifuroxazide quantitatively, and the percentage of sub-G1 cells in A375 and B16-F10 cells was detected by FCM. As shown in Supplementary Fig. S3b, the percent of sub-G1 A375 cells increased from 7.6% to 23.4% when the concentration was increased from 2.5 to 20 μM at 24 h, whereas the proportion of apoptotic cells was merely 4.2% in the vehicle control. The apoptosis rate was much higher after 48 h of treatment (Fig. 2a). Treatment of B16-F10 cells with nifuroxazide for 24 h and 48 h also resulted in significant dose-dependent apoptosis (Fig. 2a and Supplementary Fig. S3b).

Finally, we also confirmed the presence of apoptotic cells by Annexin V-FITC/PI dual-labeling by FCM. As shown in Fig. 2b and Supplementary Fig. S4a, nifuroxazide induced melanoma cell apoptosis in a time- and concentration-dependent manner, and resulted in the early apoptotic (only Annexin V positive) as well as the late apoptotic cells (Annexin V and PI-positive). After 48 h, the percentage of apoptotic cells increased from 5.9% to 39.2% when the concentration was increased from 0 to 20 μM, respectively, indicating nearly a 33.3% change. Similar results were observed in B16-F10 cells and the percentage of apoptotic cells at different concentrations is as follows: vehicle (4.5%), 2.5 μM (10.5%), 5 μM (18.3%), 10 μM (26.7%), and 20 μM (30.2%). The above data indicated that the inhibition of melanoma cells by nifuroxazide is mediated by the induction of apoptosis.

### Nifuroxazide induced apoptosis *via* the mitochondria-mediated apoptotic pathway

**Figure 3:**
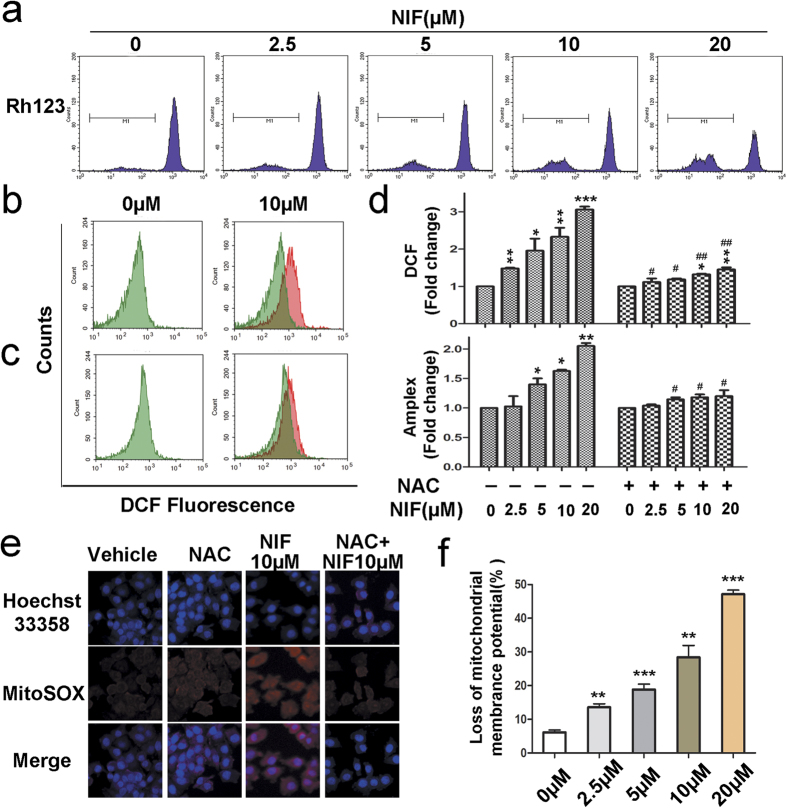
Effects of Nifuroxazide on the intrinsic apoptosis pathway. (**a**,**f**) Nifuroxazide decreased the mitochondrial membrane potential (ΔΨm) in A375 cells. A375 cells were treated with various concentrations of nifuroxazide for 24 h and then stained with 5 μg/mL Rh123 to detect the change of ΔΨm by FCM. The quantified values are also shown. A375 cells were pretreated (**c**,**d**) or not pretreated (**b**,**d**) with 2 mM NAC for 1 h and then treated with 0~20 μM nifuroxazide. The harvested cells were loaded with DCFH-DA or Amplex Red and measured by FCM or a fluorescence microplate reader. Quantification of ROS is also shown (*P < 0.05; **P < 0.01; ***P < 0.001 compared with the vehicle group; ^#^*P* < 0.05; ^##^*P* < 0.01 compared with the treated group without pretreatment with NAC). (**e**) Mitochondrial superoxide indicator (MitoSOX Red, red fluorescence) was also used to detect the ROS formation in mitochondria. The blue fluorescent staining (Hoechst 33358 staining) cells represent total cells.

To further confirm the pro-apoptotic effects of nifuroxazide, some apoptosis-related proteins were detected by western blot. Then we examined Bcl-2, Bax and cleaved caspase-3 expression levels in A375 cells after nifuroxazide treated for 24 h. As shown in Fig. 2c,d, the expression of Bcl-2 significantly decreased, whereas that of cleaved caspase-3 and Bax increased in a concentration-dependent manner and a significant increase in the ration of Bax/Bcl-2 (Fig. 2e). These results suggested that nifuroxazide-induced apoptosis might be *via* the mitochondrial apoptotic pathway. In order to verify the hypothesis, we tested the changes in the mitochondrial membrane potential (ΔΨm) by FCM using a green fluorochrome Rh123. As shown in Fig. 3a,f, a significant loss of ΔΨm was observed after nifuroxazide treatment. It is well known that mitochondria are the major source of reactive oxygen species (ROS) generation. In our experiment, ROS formation was detected by FCM or microscopy using some indicators, DCFH-DA, Amplex red and Mitosox red. The results showed that the levels of ROS increased in a dose-dependent manner after treatment with nifuroxazide (Fig. 3b,d,e), but remained unaffected when the cells were pretreated with the antioxidant NAC for 1 h (Fig. 3c–e). These results confirmed that the inhibition of melanoma cells by nifuroxazide is mediated by the induction of apoptosis through the mitochondria-mediated apoptotic pathway.

### Nifuroxazide inhibited the migration and invasion of melanoma cells

**Figure 4:**
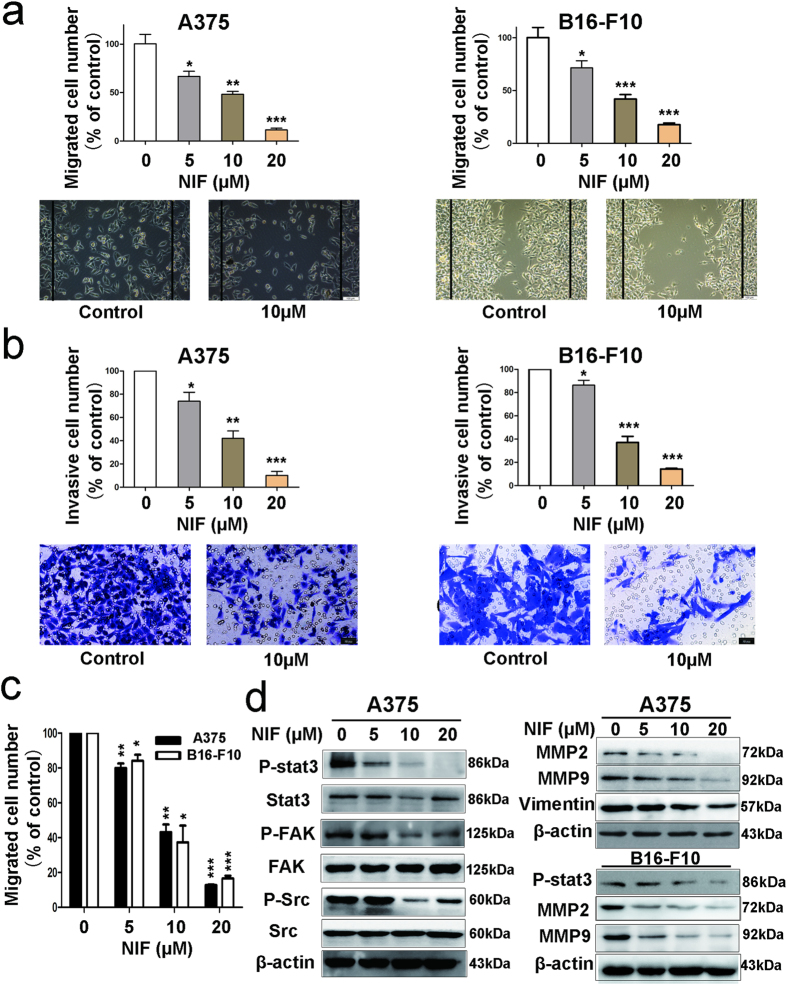
Nifuroxazide inhibits melanoma cell A375 and B16-F10 migration and invasion. (**a**,**c**) Nifuroxazide inhibited the migration of melanoma cells in wound healing assay and transwell assay. In the wound healing assay, cells were seeded in 6 well culture plates, and the cell monolayer was wounded manually. Then, the images were taken after treated with nifuroxazide for 24 h. In transwell assay, cells were seeded in the top chamber of transwell with serum-free medium and treated with various concentrations of nifuroxazide. After 24 h, the migrated cells were fixed, stained and quantified. (**b**) Nifuroxazide inhibited the invasion of melanoma cells in transwell assay. 1 × 10^5^ cells were planted in which were pre-treated with Matrigel on the upper chamber membrane and treated with indicated concentrations of nifuroxazide, and the bottom chamber was filled with the complete medium containing 10% FBS. (**d**) Western blot analysis of A375 cells following nifuroxazide treatment, including the expressions of Stat3/p-Stat3, FAK/P-FAK, Src/P-Src, MMP2, MMP9 and Vimentin.

Wound healing assays and transwell assays were used to assess the effects of nifuroxazide on melanoma cell migration and invasion, respectively. As shown in Fig. 4a, the wounding-healing assay indicated that nifuroxazide significantly inhibited the migration of both A375 and B16-F10 cells in a dose-dependent manner while the cells were found to migrate to the wound area in the vehicle group. Similar results were obtained in transwell migration assays (Fig. 4c). In addition, transwell invasion assays assessed the ability of A375 cells to invade through the matrigel; 20 μM nifuroxazide almost inhibited A375 cell invasion (Fig. 4b). Similar results were observed for B16-F10 cells. Furthermore, we investigated whether p-Stat3^Tyr705^, MMP-2, MMP-9 or other proteins, considered to be related with cell migration and invasion, are involved in nifuroxazide’s inhibitory effects on migration and invasion. The results of western blot indicated that the treatment with nifuroxazide significantly inhibited the levels of p-Stat3, MMP-2, MMP-9, vimentin, P-FAK, and P-Src in A375 cells (Fig. 4d and Supplementary Fig. S4b). Moreover, nifuroxazide treatment also decreased the expression of MMP-2, MMP-9, and p-Stat3 in B16-F10 cells (Fig. 4d and Supplementary Fig. S4b). Taken together, all of these results implied that nifuroxazide inhibited the migration and invasion of melanoma cells.

### Antitumor efficacy of nifuroxazide in xenograft model of human melanoma A375

**Figure 5:**
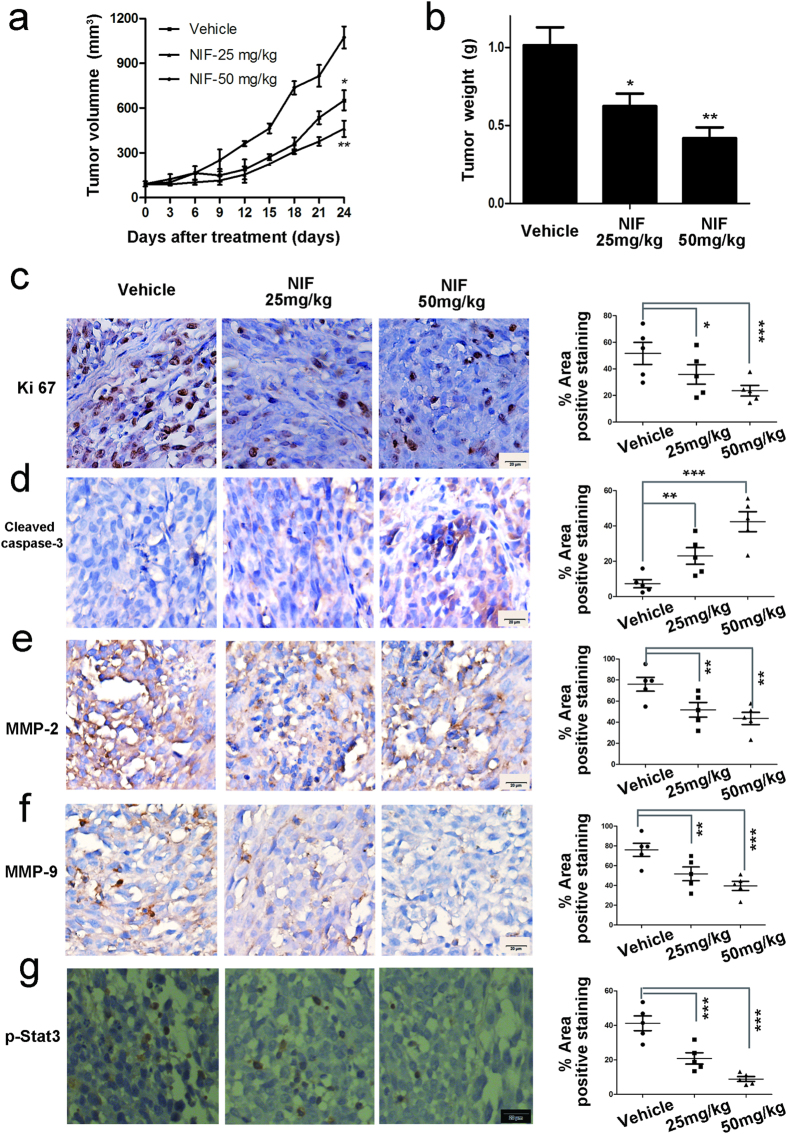
Effects of nifuroxazide on tumor growth *in vivo*. Mice implanted with A375 xenografts were treated with 25 and 50 mg/kg/day when the tumors grew to about 100 mm^3^. (**a**) Tumor volumes were measured every 3 days and presented as mean ± SD (n = 6, *P < 0.05; **P < 0.01). (**b**) The bar charts of tumor weight. (**c**) Tumor cell proliferation was evaluated through immunohistochemical analysis staining with Ki67, and the statistical data of Ki67 positive cell number were shown on the right. (**d**) The apoptosis of tumor was determined by cleaved caspase-3 immunohistochemical staining, and the statistical data of CC-3 positive cell number were shown on the right. Similarly, the immunohistochemical analysis was performed to measure the expressions of MMP-2 (**e**), MMP-9 (**f**) and p-Stat3 (**g**) in tumor tissues. *P* values for comparison of two groups were determined by 2-tailed Student’s *t* test (*P < 0.05; **P < 0.01; ***P < 0.001 vs vehicle control).

To determine whether the antitumor activity of nifuroxazide *in vivo* is consistent with its effects *in vitro*, A375-bearing mice were treated with nifuroxazide at 25 mg/kg and 50 mg/kg. As shown in Fig. 5a,b, and Supplementary Fig. S5a, compared with the vehicle group, treatment with nifuroxazide could inhibit tumor growth and tumor weight in a dose-dependent manner, with the inhibition rate of tumor volumes being 43.0% and 62.1% at 25 mg/kg and 50 mg/kg, respectively. Moreover, immunohistochemistry analyses were performed to evaluate the anti-tumor mechanism of nifuroxazide in the A375 model. As shown in Fig. 5c,d, nifuroxazide significantly inhibited the proliferation of nuclear Ki-67-positive cells and induced apoptosis cells of cleaved caspase-3-positive cells. Besides, we also found that treatment with nifuroxazide could inhibit the expression of MMP-2, MMP-9 and p-Stat3 in A375 tumor tissues (Fig. 5e,f). Overall, these data suggest that nifuroxazide inhibited melanoma tumor tissues by inhibiting cell proliferation, inducing apoptosis, and blocking metastasis, which is consistent with the *in vitro* data.

### Suppression of pulmonary metastasis of B16-F10 mouse melanoma by nifuroxazide

**Figure 6:**
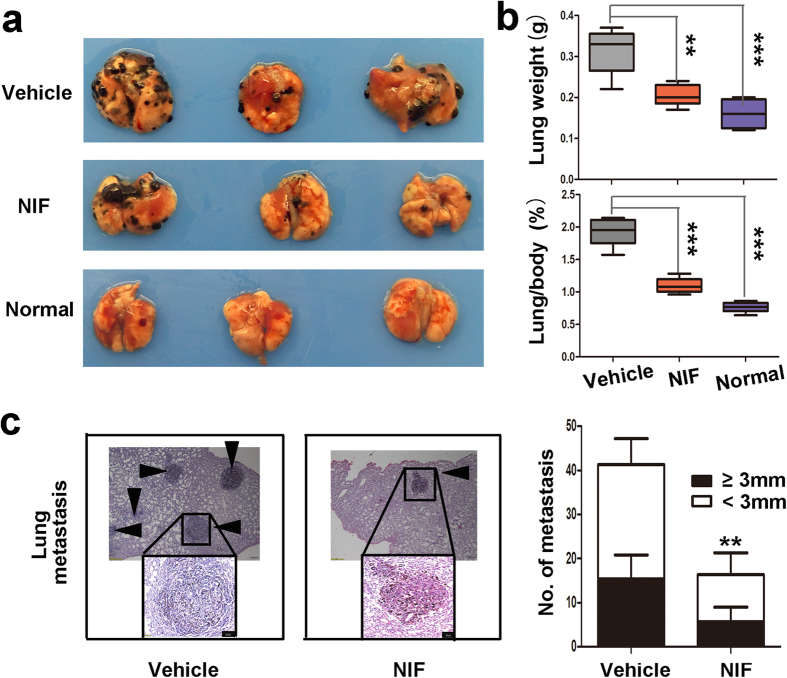
Nifuroxazide inhibited tumor metastasis. 2 × 10^5^ B16-F10 cells were injected intravenously *via* the tail vein to produce experimental lung metastasis. The mice were randomly assigned to groups on day 6 and were administrated intraperitoneally injection of nifuroxazide 50 mg/kg or vehicle once a day. (**a**) Lung metastatic nodules were visualized. (**b**) The weight of lungs and the lung/body coefficients in each group. (**c**) Metastases count at day of sacrifice in lung of vehicle and NIF-treated groups using H&E staining and macroscopic pictures. Black arrowheads represented lung metastases. *P* values for comparison of two groups were determined by 2-tailed Student’s *t* test (*P < 0.05; **P < 0.01; ***P < 0.001 vs vehicle control).

We next functionally analyzed the metastatic spread from melanoma *in vivo* by inoculating B16-F10 cells intravenously into *C57Bl/6* mice. After the mice were sacrificed, lung weight was measured, metastatic lung nodules were counted, and lung sections were stained with H&E. As shown in Fig. 6b, the lung/body coefficient decreased about two-fold after treatment with nifuroxazide compared to the observed for the vehicle group. Besides, the number of lung metastatic nodules significantly decreased in the nifuroxazide-treated mice (Fig. 6a,c). These data indicated that the treatment with nifuroxazide almost fully prevented lung metastases.

### The lung metastatic environment was modulated by nifuroxazide

**Figure 7:**
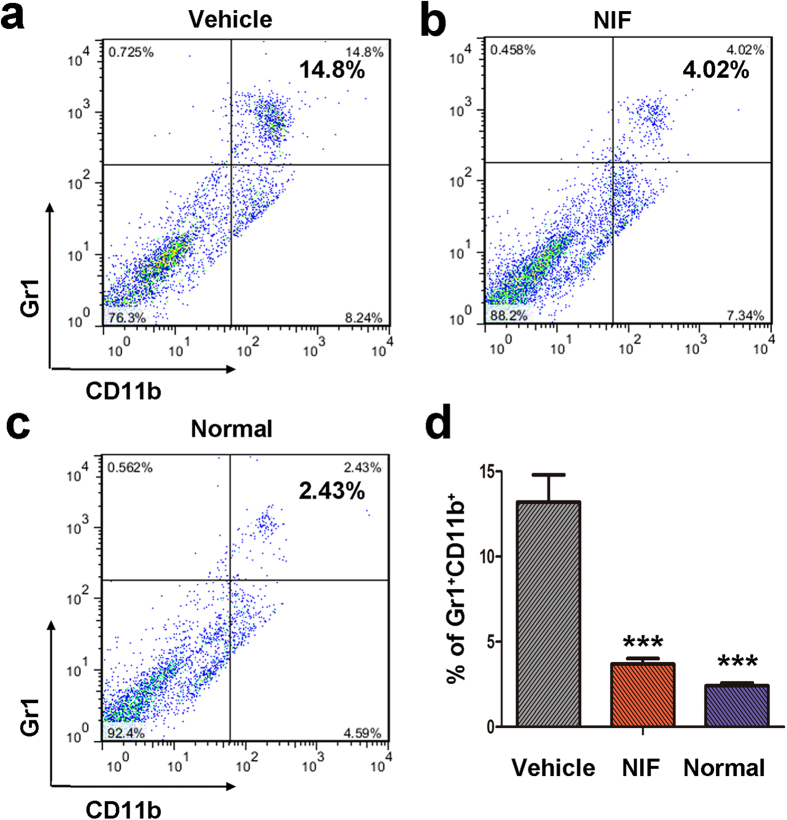
Nifuroxazide reduced MDSCs (Gr1^+^CD11b^+^) infiltration in lung sections. FCM was used to analyze the expression of MDSCs. The MDSCs were isolated from the lungs of experimental melanoma metastasis model mice treated with vehicle (**a**), or treated with nifuroxazide at 50 mg/kg (**b**), or normal mice (**c**) using as negative contrast. (**d**) Statistic results of each group were shown. Values represented mean ± SD (n = 6, ***P < 0.001).

MDSCs play an important role in stimulating B16-F10 melanoma cells *in vitro* proliferation and *in vivo* growth and metastasis^21^. We measured MDSCs, which are characterized by CD11b^+^ and Gr1^+^ double-positive myeloid cells to investigate the lung myeloid cell infiltration by FCM. After treatment with nifuroxazide for 14 days, the percentage of MDSCs decreased in the nifuroxazide-treated group as compared with the control group in a concentration-dependent manner (Fig. 7a–c). Furthermore, statistical analysis also demonstrated that about 3-fold reduction of MDSCs in the lung was observed after 50 mg/kg nifuroxazide treatment (Fig. 7d). These data suggested that nifuroxazide inhibited the infiltration of MDSCs into the lung, which might be associated with suppression of distant colonization of tumor cells in B16-F10 melanoma metastasis model.

### Toxicity evaluation

To elucidate the safety profile of nifuroxazide, all mice in A375 and B16-F10 models were sacrificed after treatment with nifuroxazide followed by toxicity evaluation, including serological analysis, hematological analysis and H&E staining. Serological and hematological analysis did not show any pathological changes and no significant changes in body weights were observed relative to the vehicle group (Supplementary Fig. S5b, Supplementary Fig. S6, and Supplementary Fig. S7). Moreover, microscopic examination of the heart, liver, spleen and kidney showed no pathologic changes after treatment with nifuroxazide (Supplementary Fig. S6, and Supplementary Fig. S7).

## Discussion

Melanoma is highly malignant with considerable metastatic potential and drug resistance, and the incidence is increasing rapidly^3^. As one of the deadliest form of skin cancers, there is an urgent need to discover novel potential drug candidate against melanoma^1^,^11^. Recent studies have reported that Stat3 is activated at 50–90% frequency in diverse cancers, including melanoma^23^. Stat3 activity increased melanoma invasiveness and is required for active melanogenesis by regulating tyrosinase gene expression and enzyme activity. Besides, blockade of Stat3 by inhibitors could inhibit angiogenesis and trigger apoptosis in melanoma cells^24^,^25^. Therefore, directly inhibit Stat3 might be a promising novel approach for melanoma therapy.

In this study, nifuroxazide, an oral anti-diarrheal agent identified as an inhibitor of Stat3, was evaluated for its potency against melanoma *in vitro* and *in vivo*. The MTT assay showed that nifuroxazide inhibited the proliferation of melanoma cells in a time- and dose-dependent manner, and colony formation assay was performed to visually confirm this result. To investigate the mechanism underlying the anti-proliferation properties of nifuroxazide, cell cycle analysis was performed and, nifuroxazide was found to induce G2/M phase arrest in A375 and B16-F10 cells. Cell cycle deregulation plays an important role in modulating cell proliferation, and is tightly regulated by the cyclin/cyclin-dependent kinase (CDK) complexes^30^. In the present study, treatment of A375 cells with nifuroxazide decreased the protein levels of cyclin B1 and P-cdc25c and increased the expression of P-CDK1. We might conclude that nifuroxazide caused G2/M arrest by decreasing the formation of cyclin B1/CDK1 complex via inhibition of cyclin B1 and dephosphorylation of CDK1 at Tyr15, an activity that is associated with the decreased level of cdc25c.

Apoptosis is a major route to eradicate cancer cells and involves cell cycle arrest^31^. Among the apoptosis-related proteins, caspase-3 is activated by upstream effector proteins, leading to apoptosis cascade^32^. Mitochondria play an important role in the intrinsic apoptosis pathway by altering the mitochondrial transmembrane potential; the intrinsic apoptosis pathway involves the participation of Bcl-2 family proteins, including the anti-apoptotic protein Bcl-2 and the pro-apoptotic protein Bax^33^. In this work, Hoechst 33358 staining and FCM assays both revealed that nifuroxazide treatment induced apoptosis in melanoma cells in a time- and concentration-dependent manner. In addition, the activation of cleaved-caspase-3 was observed after treatment with nifuroxazide. Moreover, the occurrence of apoptosis was associated with the activation of Bax and down-regulation of Bcl-2, which also induced a loss of ΔΨm in A375 cells after nifuroxazide treatment. Further, we found that nifuroxazide significantly increased ROS production in A375 cells, while pre-treatment with the ROS inhibitor NAC partially abrogated the nifuroxazide-induced increase of ROS. That is consistent with our result of the dose-dependent decrease in the mitochondria membrane potential. Therefore, this data indicated that nifuroxazide treatment induced apoptotic death in melanoma through ROS-medicated mitochondrial apoptotic pathway.

Moreover, in our established A375 tumor model in BALB/c athymic mice, the tumor growth was inhibited by nifuroxazide administration (50 mg/kg/d) with an inhibitory rate of 62.1%. Meanwhile, more cleaved caspase-3-positive cells and fewer cells Ki67-positive and p-Stat3-positive cells were observed in the tumor tissues treated with nifuroxazide than in the control group, indicating that nifuroxazide could inhibit cell proliferation and induce cell apoptosis through decreased the expression of p-Stat3 *in vivo*.

Melanoma is a disease that could spread to the lung, lymph nodes or other organs^34^. In addition, the poor efficacy against melanoma could be attributed to high metastatic potential of melanoma cells^35^. Therefore, the inhibitory effects of nifuroxazide on metastasis were evaluated by cell invasion and migration assays. The transwell assay indicated that nifuroxazide inhibited the migration and invasion activities of A375 and B16-F10 cells. Moreover, some cell invasion- and migration-related, such as p-stat3^Tyr705^, MMP-2, MMP-9, vimentin, P-FAK, and P-Src, were down regulated by nifuroxazide. Similar results were observed in the A375-bearing mice model, with reduction in the number of MMP-2 and 9-positive metastasis cells, as analyzed by immunohistochchemistry.

Next, we used the B16-F10 melanoma lung metastasis model to further verify this result. The number of lung metastatic nodules, the lung/body coefficient and H&E staining indicated that nifuroxazide prevented the generation of lung metastases. Recent studies showed that lysosomal acid lipase play a critical role in regulating MDSCs (CD11b^+^/Gr1^+^) stimulate cancer cell proliferation and overcome cancer metastasis through modulation of the mTOR pathway, which provides a mechanistic basis for targeting MDSCs to reduce the risk of cancer metastasis^29^. Our *in vivo* studies indicated that the treatment with nifuroxazide caused a significant decrease in the number of CD11b^+^/Gr1^+^ in lungs compared with that of the untreated groups. It is therefore conceivable that nifuroxazide can inhibit lung metastasis by reducing the number of MDSCs in lung.

In conclusion, we have assessed the anti-melanoma activities of nifuroxazide *in vitro* and *in vivo*. Mechanism studies showed that nifuroxazide could arrest cell cycle in G2/M phase and induce cell apoptosis *via* ROS-mitochondrial apoptotic pathway. In addition, we found that nifuroxazide markedly impaired melanoma cell migration and invasion. Moreover, nifuroxazide showed anti-tumor activities in the melanoma xenograft *in vivo* without obvious toxicity. Importantly, nifuroxazide also inhibited lung metastasis by reducing the number of MDSCs in lung. Therefore, this study indicated that nifuroxazide is a potential agent for inhibiting melanoma growth and metastasis.

## Materials and Methods

### Regents and preparation of nifuroxazide

MTT, DMSO, DCFH-DA, Rh123, Cremophor EL and PI were purchased from Sigma Chemical Co. (St Louis, MO, USA). NAC and Hoechst33358 were obtained from Beyotime (Beijing, China). MitoSOX red and Amplex red were purchased from Yeasen (Shanghai, China) and Invitrogen (Carlsbad, USA), respectively. The Annexin V-FITC apoptosis detection kit was purchased from KeyGen Biotech (Nanjing, China). The primary antibodies were obtained from Cell Signaling Technology Company (Beverly, MA). FITC-CD11b, and PE-Gr1 conjugated antibodies were acquired from BD Biosciences.

Nifuroxazide was purchased from Xiyashiji Chemical Co. Ltd (Chengdu, Sichuan, China), and was determined by ^1^H-NMR, ^13^C-NMR and ESI-MS analysis. For the *in vitro* studies, nifuroxazide was prepared in DMSO at a stock concentration of 20 mM and diluted in the relevant medium at final DMSO concentration of 0.1% (*V/V*), and medium with 0.1% DMSO served as vehicle control. For *in vivo* experiment, nifuroxazide was dissolved in 25% (v/v) Cremophor EL/ethanol (50:50, Sigma Cremophor EL, 100% ethyl alcohol) and 75% ultrapure water.

### Cell lines and cell culture

Human melanoma cells A375, A2058 and A875, non-cancerous cells HEK293, as well as murine melanoma cells B16-F10 were purchased from American Type Culture Collection (ATCC, Manassas, VA, USA). All of them were cultured in DMEM or RPIM 1640 medium, containing 10% fetal bovine serum (FBS, Gibco, Auckland, N.Z.), 4 mM L-Glutamine, penicillin-streptomycin (Life Technologies), and cultured in a humidified atmosphere under 5% CO_2_ at 37 °C.

### Cell proliferation assay

Briefly, cells (2–4 × 10^3^/100 μL) were seeded in 96-well microplates, and treated with nifuroxazide for 24, 48 or 72 h. Then, 20 μL of the MTT solution (5 mg/mL) was added to each well and incubated for another 1–4 h at 37 °C. The formazan crystal formed by the living cells was dissolved with 150 μL of DMSO. After 10 min, the optical density was measured using Spectra MAX M5 microplate spectrophotometer (Molecular Devices) at 570 nm. This assay was done on all the 4 melanoma cell lines, A375, A2058, A875, and B16-F10.

### Colony formation assay

Cells were assayed for colony formation ability by replating them in specified numbers (400–1000 cells/well) in a six-well plate, and treating with various concentrations of nifuroxazide (0–2.5 μM). Then, the cells were incubated once every 3 days for additional 13 days and stained with 0.5% crystal violet in absolute ethanol after fixing. This assay was done on all the 4 melanoma cell lines, A375, A2058, A875, and B16-F10. Unless otherwise stated, the following assays were done on A375 and B16-F10 cell lines.

### Morphological analysis by Hoechst staining

To detect the apoptosis induction effect of nifuroxazide, we analyzed the morphological changes associated with apoptosis by Hoechst33358 staining. After incubating with nifuroxazide for 24 h, the cells were fixed with paraformaldehyde and stained with Hoechst33358 solutions (5 μg/mL). Then, the nuclear morphology of cells was observed by fluorescence microscopy (Zeiss, Axiovert 200, Germany).

### Cell cycle and apoptotic assays by FCM

The cell cycle distribution was analyzed by FCM as described previously with slight modification^36^. Briefly, the cells were treated with nifuroxazide and fixed with 75% ethanol overnight. The cells were suspended in 500 μL hypotonic solution containing 50 μg/mL PI, 0.1% sodium citrate and 0.1% Triton X-100 in the dark, and then analyzed by FCM (Becton Dickinson, USA). Data were analyzed using Modfit 2.8 software.

To further investigate the apoptosis-induction effects of nifuroxazide, apoptotic cells were detected by FCM as described previously with slight modifications^36^. After treatment with nifuroxazide, the harvested cells were stained with PI solution or Annexin V-FITC/PI detection kit according to the manufacturer’s instructions, and detected using a flow cytometer (Becton Dickinson, USA). Finally, the data was analyzed by Flow Jo software.

### Measurement of ROS levels in cells

After exposure to different concentrations of nifuroxazide for 24 h, A375 cells were incubated with DCFH-DA (10 μM), MitoSOX (5 μM) or Amplex red (50 μM) at 37 °C for about 20 min^37^,^38^. Direct imaging of ROS in probe-loaded cells was performed on the Thermo Scientific Cellomics^®^ Array Scan^®^ VT1 (Thermo, USA). Alternatively, the fluorescence intensity was measured by FCM or Spectra MAX M5 microplate spectrophotometer (Molecular Devices). To further confirm the changes in ROS, antioxidant treatment by 2 mM N-acetyl-L-cysteine (NAC, a ROS inhibitor) for 1 h was done prior to nifuroxazide exposure.

### Mitochondrial membrane potential (ΔΨm) assay

2-(6-Amino-3-imino-3H-xanthen-9-yl) benzoic acid methyl ester (Rh123) was used to determine the changes in ΔΨm by FCM^39^. After the treatment with nifuroxazide for 24 h, the harvested cells were incubated with Rh123 solution (5 μg/mL) at 37 °C for 30 min in the dark and ΔΨm was then measured by FCM.

### Scratch-induced migration assay *in vitro*

When monolayer cells grew to about 80% confluence, they were wounded by the tip of a sterile 200-μL micropipette. After incubation with nifuroxazide for 24, images were taken by a microscope (Zeiss, Axiovert 200, Germany). The migrated cells were quantified by manual counting.

### Boyden chamber migration and invasion assay

Modified transwell invasion assay was conducted as described previously with minor modifications^40^. Briefly, Matrigel (BD Biosciences, USA) diluted 1:3 in serum-free medium was added (60 μL/well) to the upper surface of 24-well transwell plate (Millipore). After Matrigel polymerization, the top of the Matrigel layer was seeded with 1 × 10^5^ A375 cells or B16-F10 cells in 100 μL serum-free medium to which with 0.1% DMSO or various concentrations of nifuroxazide were added, and the lower compartments were filled with 600 μL of the complete medium, which served as a chemoattractant. After 24 h, the cells on the top were moved with a cotton swab, and the invasion cells on the filters were stained with crystal violet after fixed with 4% paraformaldehyde. The images were taken using a Zeiss digital microscope. Five independent areas per well were counted and the mean number of migrated cells was calculated. Boyden chamber migration assay was performed according to previous studies^41^. 1 × 10^5^ A375 cells or B16-F10 cells in 100 μL serum-free medium were added to the upper chamber, and the bottom chamber was filled with 600 μL of the complete medium containing 10% FBS. Both chambers were added with different concentrations of nifuroxazide. After 24 h, a cotton swab was used to discard the non-migrated cells in the upper chamber, and the migrated cells were stained with 0.5% crystal violet. In total, 5 random fields were counted and photographed under a light microscope.

### Western blot analysis

To determine the effect of nifuroxazide on relevant signaling pathway, some proteins in A375 or B16-F10 cells were evaluated using western blot. A375 or B16-F10 cells were incubated with indicated concentration of nifuroxazide for 24 h. Harvested cells were lysed in RIPA buffer (Beyotime, Beijing, China) for 30 min and equalized before loading. The samples were separated on SDS-PAGE gel and transferred onto polyvinylidene fluoride (PVDF) membranes (Amersham Bioscience, Piscataway, NJ). After incubated with primary and secondary antibodies, the immunoreactive protein bands were detected using the enhanced chemiluminescence kit (Millipore, USA). A monoclonal β-actin antibody was used as a control.

### Mice and tumor model

All animal experiments have been approved by the Institutional Animal Care and Treatment Committee of Sichuan University in China (Permit Number: 20150409) and were carried out in accordance with the approved guidelines. Mice used in this study were obtained from Beijing HFK bioscience CO. LTD, Beijing, China and were maintained in a specific-pathogen-free (SPF) condition facility with an air-conditioned room at 25 ± 2 °C with a relative humidity of 40–70%, and a 12-h light/dark cycle. Mice engrafted subcutaneously with 1 × 10^7^ A375 cells were randomly divided into groups when tumor volume was around 100 mm^3^ and were administrated intraperitoneally injected with nifuroxazide 25 mg/kg, 50 mg/kg or vehicle once daily. The tumor size and body weight were measured every 3 days. The mice were sacrificed at an endpoint defined by the tumor volume (~1,000 mm^3^). Tumor volume is calculated as follows: Volume = 0.5 × a × b^2^, where a (mm) was the length and b (mm) is the width of the tumor.

### Experimental melanoma metastasis model

C57Bl/6J mice were engrafted by injecting intravenously *via* the tail vein with 2 × 10^5^ B16-F10 cells to produce experimental lung metastasis. They were randomly assigned to groups on day 6 and were intraperitoneally injected with nifuroxazide 50 mg/kg or vehicle once daily. Another group of normal animals without injected cells was used as control animals. All mice (both experimental and control groups) were killed on day 21, followed by a thorough visual examination of all internal organs for visible melanotic nodules. Black dots on lung surface were counted and confirmed as melanoma metastases^42^. About 1 × 10^6^ fresh single-cell suspensions from lung sections were prepared in PBS and were labeled with fluorescence-conjugated antibodies. We collected the fluorescence data from FCM (BD Biosciences) and analyzed them using Flow Jo software^16^.

### Immunohistochemistry

Immunohistochemistry (IHC) staining of tumors and lung sections was described previously^16^. Paraffin-embedded tumor sections were stained with primary antibodies (Ki67, cleaved caspase-3, MMP-2, MMP-9, and p-Stat3) using the DAB Detection Kit. Besides, paraffin lung sections were stained with H&E.

### Toxicity evaluation

To investigate the safety profile of nifuroxazide in rats, the blood obtained from eyeball was used for serological and hematological analysis when the mice were sacrificed. Mouse tissue samples (heart, liver, spleen and kidney) were fixed in 4% paraformaldehyde and embedded in paraffin. These samples were processed into sections of 4-μm-thick sections and the slides were stained with hematoxylin and eosin (H&E) according to standard protocols.

### Statistical analysis

Cell culture-based experiments were done at least in biological triplicates. Quantifications of staining were done on sections of at least three different views. *P* value for comparison of two groups were determined by 2-tailed Student’s *t* test. *P* value < 0.05 was considered statistically significant.

## Additional Information

**How to cite this article**: Zhu, Y. *et al*. Nifuroxazide exerts potent anti-tumor and anti-metastasis activity in melanoma. *Sci. Rep*. **6**, 20253; doi: 10.1038/srep20253 (2016).

## Supplementary Material

Supplementary Information
