# Author Correction: Nifuroxazide exerts potent anti-tumor and anti-metastasis activity in melanoma

**DOI:** 10.1038/s41598-022-14642-8

**Published:** 2022-06-28

**Authors:** Yongxia Zhu, Tinghong Ye, Xi Yu, Qian Lei, Fangfang Yang, Yong Xia, Xuejiao Song, Li Liu, Hongxia Deng, Tiantao Gao, Cuiting Peng, Weiqiong Zuo, Ying Xiong, Lidan Zhang, Ningyu Wang, Lifeng Zhao, Yongmei Xie, Luoting Yu, Yuquan Wei

**Affiliations:** 1 State Key Laboratory of Biotherapy/Collaborative Innovation Center for Biotherapy, West China Hospital, West China Medical School, Sichuan University, Chengdu, 610041 Sichuan China; 2 College of Agricultural and Life Sciences, University of Wisconsin-Madison, Madison, WI 53706 USA; 3 Department of Pharmaceutical and Bioengineering, School of Chemical Engineering, Sichuan University, Chengdu, 610041 Sichuan China; 4 Department of Pharmacy, Xinqiao Hospital, Third Military Medical University, Chongqing, 404100 China

Correction to: *Scientific Reports* 10.1038/srep20253, published online 02 February 2016

This Article contains errors in Figure 5.

As a result of an error in figure assembly, the representative image provided for NIF 25 mg/kg of Figure 5(e) is incorrect. The dot plot of Figure 5(f) has also been inadvertently duplicated from Figure 5(e). The correct Figure 5 and accompanying legend appear below.

**Figure 5:**
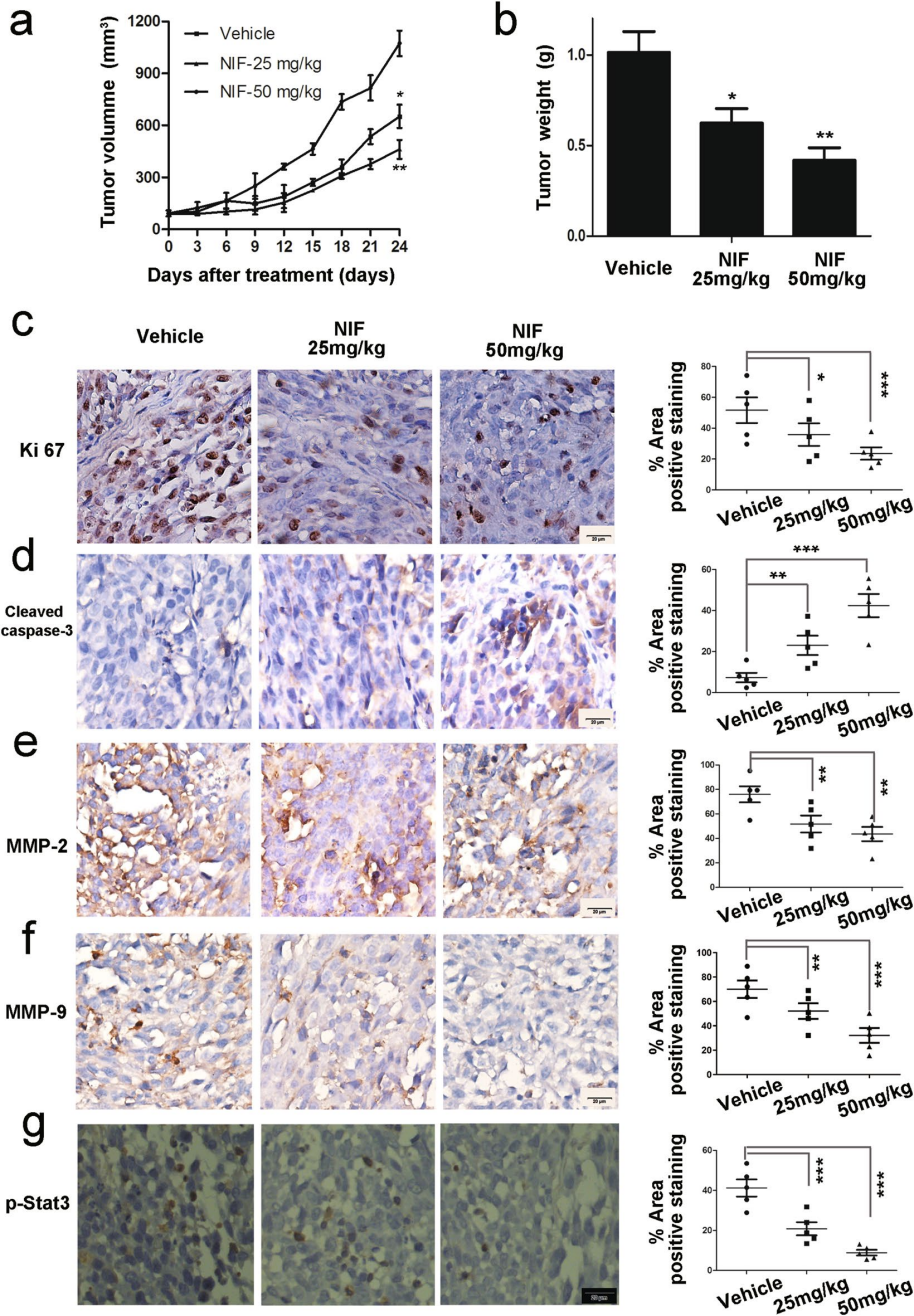
Effects of nifuroxazide on tumor growth in vivo. Mice implanted with A375 xenografts were treated with 25 and 50 mg/kg/day when the tumors grew to about 100 mm3. (**a**) Tumor volumes were measured every 3 days and presented as mean ± SD (n = 6, *P < 0.05; **P < 0.01). (**b**) The bar charts of tumor weight. (**c**) Tumor cell proliferation was evaluated through immunohistochemical analysis staining with Ki67 and the statistical data of Ki67 positive cell number were shown on the right. (**d**) The apoptosis of tumor was determined by cleaved caspase-3 immunohistochemical staining and the statistical data of CC-3 positive cell number were shown on the right. Similarly, the immunohistochemical analysis was performed to measure the expressions of MMP-2 (**e**), MMP-9 (**f**) and p-Stat3 (**g**) in tumor tissues. *P* values for comparison of two groups were determined by 2-tailed Student’s t test (*P < 0.05; **P < 0.01; ***P < 0.001 vs vehicle control).

These changes do not affect the conclusions of the Article.

