# Supplementary material for: Nifuroxazide exerts potent anti-tumor and anti-metastasis activity in melanoma
Source: Sci Rep. 2016 Feb 2;6:20253. doi: 10.1038/srep20253 (PMC4735744; doi:10.1038/srep20253)
Supplement: Supplementary Information [file srep20253-s1.doc]

**Supplementary information**

**Nifuroxazide exerts** **potent anti-tumor and anti-metastasis activity in melanoma**

Yongxia Zhu^1^,**^+^**, Tinghong Ye^*,1^,**^+^**, Xi Yu^2^, Qian Lei^1^, Fangfang Yang^1^, Yong Xia^1^, Xuejiao Song^1^, Li Liu^1,3^, Hongxia Deng^1^, Tiantao Gao^1^, Cuiting Peng^1,3^, Weiqiong Zuo^1^, Ying Xiong^4^, Lidan Zhang^1^, Ningyu Wang^1^, Lifeng Zhao^1^, Yongmei Xie^1^, Luoting Yu^*,1^, Yuquan Wei^1^.

^1^State Key Laboratory of Biotherapy/ Collaborative Innovation Center for Biotherapy, West China Hospital, West China Medical School, Sichuan University, Chengdu, 610041, Sichuan, China.

^2^College of agricultural and life sciences, University of Wisconsin-Madison, Madison, WI53706,USA.

^3^Department of Pharmaceutical and Bioengineering, School of Chemical Engineering, Sichuan University, Chengdu, 610041, Sichuan, China.

^4^ Department of Pharmacy, Xinqiao Hospital, Third Military Medical University, Chongqing, 404100, China.

**^+^**These authors contributed equally to this work.

^*^Corresponding author: Tinghong Ye or Luoting Yu. State Key Laboratory of Biotherapy/Collaborative Innovation Center for Biotherapy, West China Hospital, West China Medical School, Sichuan University, No. 37, Guoxue Alley, Chengdu 610041, Sichuan, China. Tel: +86 28 8516 4063; Fax: +86 28 8516 4060; E-mail: [yeth1309@scu.edu.cn](mailto:yeth1309@scu.edu.cn) or [yuluot@scu.edu.cn](mailto:yuluot@scu.edu.cn)

**Supplementary Figure S1-S7**

**Supplementary Figure S1**

**
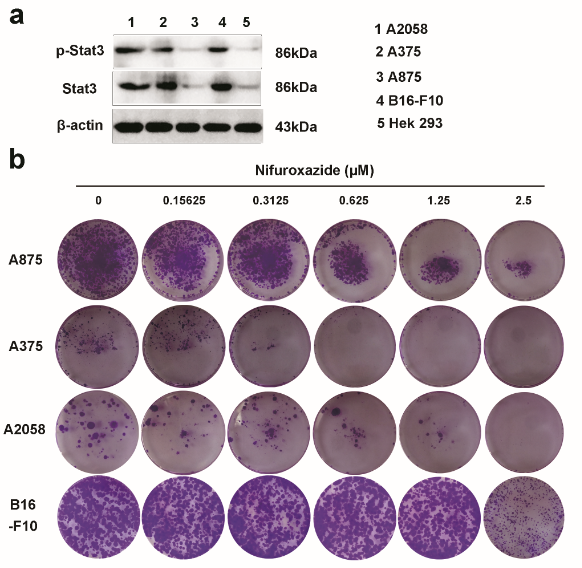
**

**Supplementary Figure 1. The effects of nifuroxazide on colony formation in melanoma cells.** (**a**) Equal protein amounts from four melanoma cancer cell lines and one non-cancerous cell line Hek293 were analyzed by western blot for phosphorylated Stat3 (p-Stat3) expression levels. (**b**) Melanoma cells A375, A2058, 875 and B16-F10 were replanted in a six-well plate, and treated with various concentrations of nifuroxazide (0-2.5 µM). After about two weeks, the cells were fixed and stained with 0.5% crystal violet.

**Supplementary Figure S2**


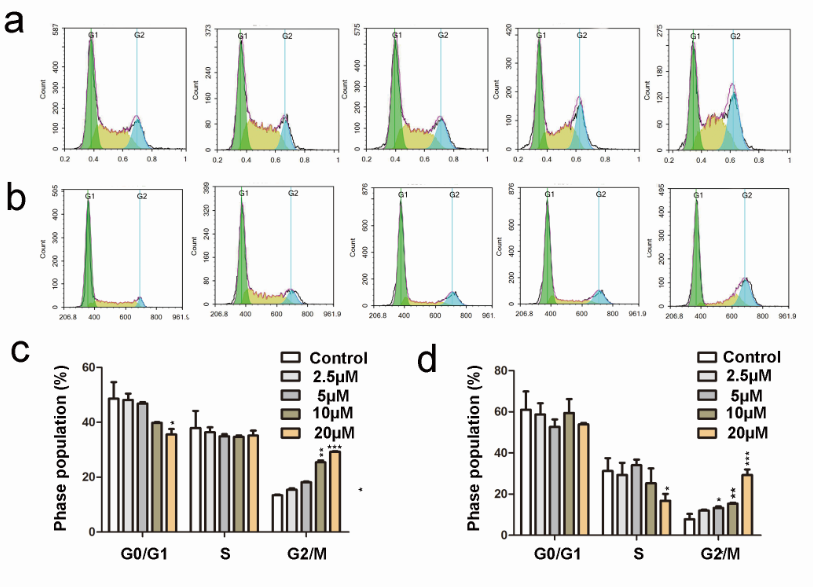


**Supplementary Figure 2 Nifuroxazide induced G2/M phase arrest in melanoma cells.** A375 (**a**) and B16-F10 (**b**) cells were incubated with nifuroxazide for 24h, and subjected to cell cycle analysis by flow cytometry after incubated with a PI solution. The cell cycle distributions in A375 (**c**) and B16-F10 (**d**) cells were displayed in quantified histograms.

**Supplementary Figure S3**


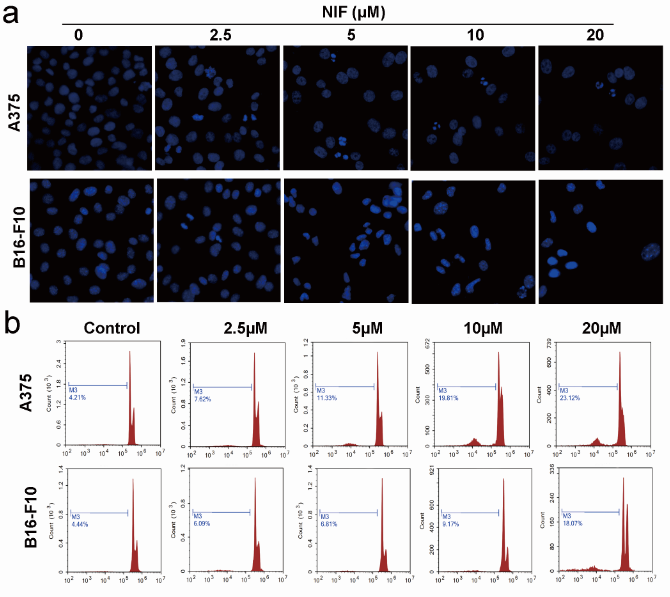


**Supplementary Figure 3 nifuroxazide induced apoptosis of A375 and B16-F10 cells.** (**a**) The fluorescence microscopic appearance of Hoechst 33358-stained A375 and B16-F10 cells after incubated with nifuroxazide for 24 h. (**b**) Flow cytometric analysis of PI-stained melanoma cell lines treated with indicated concentrations of nifuroxazide for 24 h.

**Supplementary Figure S4**


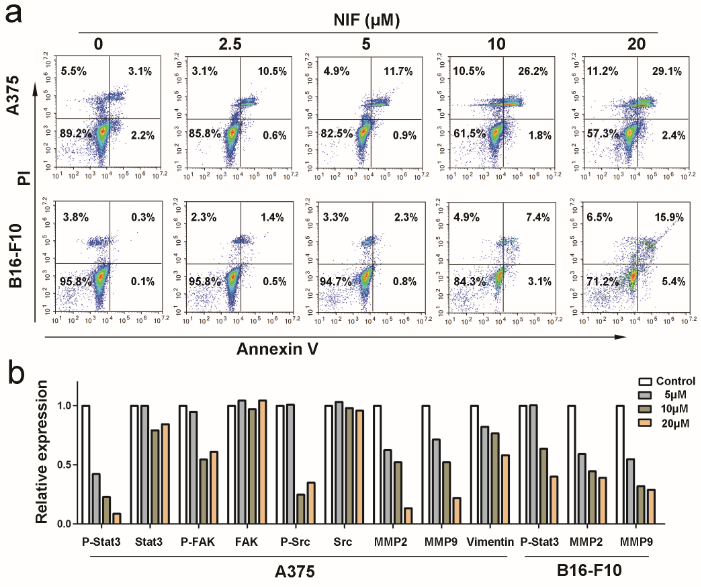


**Supplementary Figure S4** (**a**) A375 and B16-F10 cells were incubated with nifuroxazide for 24 h. The apoptosis cells stained with Annexin V-FITC/PI were measured by FCM. (**b**) Protein expressions were quantified and normalized against β-actin expression.

**Supplementary Figure S5**

**
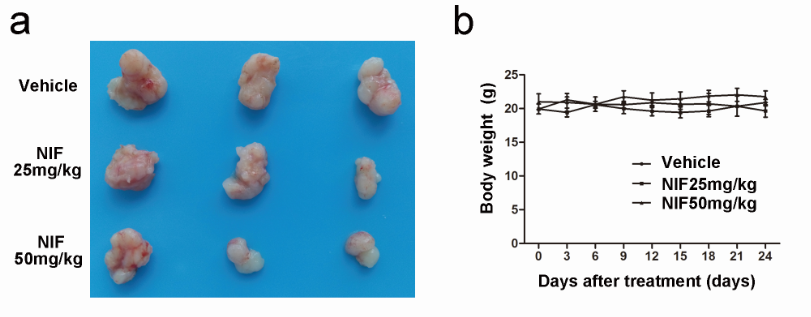
**

**Supplementary Figure S5 Nifuroxazide inhibited tumor growth in A375 model.** (**a**) After treated with nifuroxazide for 24 days, the mice were sacrificed and the subcutaneous tumors in each group were taken as a picture. (**b**) The body weight had no significant difference in A375-bearing model.

**Supplementary Figure S6**


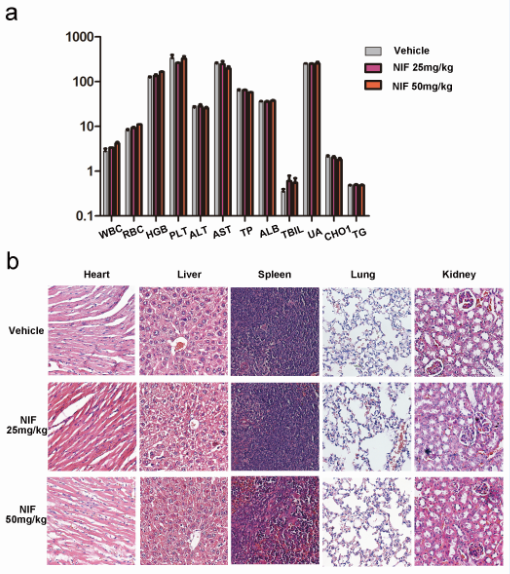


**Supplementary Figure 6 The safety evaluation of nifuroxazide in A375 xenograft model.** (**a**) After the treatment of A375 xenograft in nude mice, hematological parameters and serum biochemical values of mice were measured. Units of the parameters are as follows: WBC, PLT, 10^9^/L; RBC, 10^12^/L; HGB, TP, ALB, g/L; ALT, AST, U/L; UA, uM; TG, CHO1, mM. (**b**) H&E staining of paraffin-embedded sections of the heart, liver, spleen, lung and kidney (200 ×) after the mice were sacrificed.

**Supplementary Figure S7**


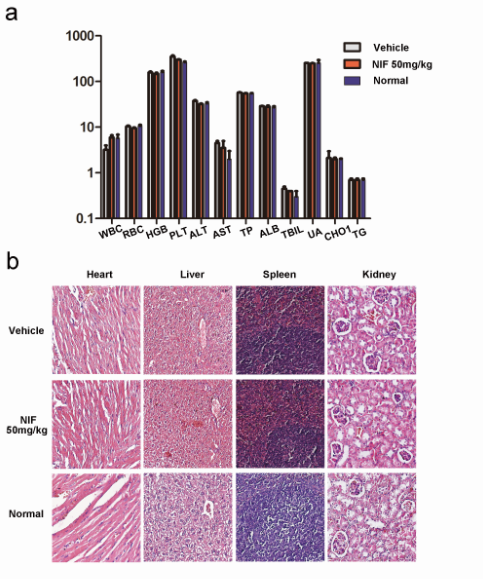


**Supplementary Figure 7 The safety evaluation of nifuroxazide in melanoma metastasis model.** (**a**) When the C57Bl/6J mice were sacrificed, hematological parameters and serum biochemical values were measured. Units of the parameters are as follows: WBC, PLT, 10^9^/L; RBC, 10^12^/L; HGB, TP, ALB, g/L; ALT, AST, U/L; UA, uM; TG, CHO1, mM. (**b**) H&E staining of paraffin-embedded sections of the heart, liver, spleen and kidney (200 ×).
